# Myosteatosis as a Shared Biomarker for Sarcopenia and Cachexia Using MRI and Ultrasound

**DOI:** 10.3389/fresc.2022.896114

**Published:** 2022-05-30

**Authors:** Jevin Lortie, Benjamin Rush, Katie Osterbauer, T. J. Colgan, Daiki Tamada, Sujay Garlapati, Toby C. Campbell, Anne Traynor, Ticiana Leal, Viharkumar Patel, Jeffrey J. Helgager, Kenneth Lee, Scott B. Reeder, Adam J. Kuchnia

**Affiliations:** ^1^Department of Nutritional Sciences, University of Wisconsin-Madison, Madison, WI, United States; ^2^Department of Radiology, University of Wisconsin-Madison School of Medicine and Public Health, Madison, WI, United States; ^3^Department of Medicine, University of Wisconsin-Madison School of Medicine and Public Health, Madison, WI, United States; ^4^Department of Hematology and Medical Oncology, Emory University School of Medicine, Atlanta, GA, United States; ^5^Department of Pathology, Harvard Medical School, Boston, MA, United States; ^6^Department of Pathology, University of Wisconsin-Madison School of Medicine and Public Health, Madison, WI, United States

**Keywords:** proton density fat fraction, echo intensity, ultrasound, MRI, cancer, elasticity, muscle quality, muscle health

## Abstract

**Purpose:**

Establish bedside biomarkers of myosteatosis for sarcopenia and cachexia. We compared ultrasound biomarkers against MRI-based percent fat, histology, and CT-based muscle density among healthy adults and adults undergoing treatment for lung cancer.

**Methods:**

We compared ultrasound and MRI myosteatosis measures among young healthy, older healthy, and older adults with non-small cell lung cancer undergoing systemic treatment, all without significant medical concerns, in a cross-sectional pilot study. We assessed each participant's rectus femoris ultrasound-based echo intensity (EI), shear wave elastography-based shear wave speed, and MRI-based proton density fat-fraction (PDFF). We also assessed BMI, rectus femoris thickness and cross-sectional area. Rectus femoris biopsies were taken for all older adults (*n* = 20) and we analyzed chest CT scans for older adults undergoing treatment (*n* = 10). We determined associations between muscle assessments and BMI, and compared these assessments between groups.

**Results:**

A total of 10 young healthy adults, 10 older healthy adults, and 10 older adults undergoing treatment were recruited. PDFF was lower in young adults than in older healthy adults and older adults undergoing treatment (0.3 vs. 2.8 vs. 2.9%, respectively, *p* = 0.01). Young adults had significantly lower EI than older healthy adults, but not older adults undergoing treatment (48.6 vs. 81.8 vs. 75.4, *p* = 0.02). When comparing associations between measures, PDFF was strongly associated with EI (ρ = 0.75, *p* < 0.01) and moderately negatively associated with shear wave speed (ρ = −0.49, *p* < 0.01) but not BMI, whole leg cross-sectional area, or rectus femoris cross-sectional area. Among participants with CT scans, paraspinal muscle density was significantly associated with PDFF (ρ = −0.70, *p* = 0.023). Histological markers of inflammation or degradation did not differ between older adult groups.

**Conclusion:**

PDFF was sensitive to myosteatosis between young adults and both older adult groups. EI was less sensitive to myosteatosis between groups, yet EI was strongly associated with PDFF unlike BMI, which is typically used in cachexia diagnosis. Our results suggest that ultrasound measures may serve to determine myosteatosis at the bedside and are more useful diagnostically than traditional weight assessments like BMI. These results show promise of using EI, shear wave speed, and PDFF proxies of myosteatosis as diagnostic and therapeutic biomarkers of sarcopenia and cachexia.

## Introduction

Debilitating muscle health conditions like sarcopenia and cachexia reduce muscle function, quality of life, and increase the risk of death (1, 2). Both conditions are generally underdiagnosed and are expected to become more prevalent with an increasing aging population (3–5). In addition, no standard definition exists for either condition due in part to the broad availability of multiple bioimaging techniques available to assess body composition and muscle health, and the limited validity of biomarkers from these techniques in clinical populations (3, 6, 7). The progressive strength and muscle mass loss that exemplifies sarcopenia is prevalent in up to 33% of older adults, leading to decreased mobility, quality of life, and an increased risk of death (3, 8, 9). Although of great consequence, the diagnosis of sarcopenia is inconsistent (3).

Similar in ambiguity, cachexia is typically an acute rapid loss of muscle and fat mass that occurs in disease states such as cancer. Although definitions vary, diagnosis typically takes into account weight loss, appetite, and BMI (10–12). Regardless of definition, the presence of cachexia in patients greatly increases the risk of death (4). Among patients with lung cancer, cachexia is prevalent in up to 61% of patients and accounts for 20% of lung cancer deaths (7). Cachexia also contributes to increased chemotherapy toxicity, reduced anti-tumor treatment effectiveness, and the increased risk of mortality (7).

The treatment of sarcopenia and cachexia would benefit from the use of accurate and consistently used, non-invasive imaging biomarkers that assess muscle health. Potential imaging assessment methods include computer tomography (CT), magnetic resonance imaging (MRI), and ultrasound (US) (13, 14). These methods can be separated into non-beside measures (CT and MRI), and bedside measures (US) each with relative advantages and disadvantages. Due to the use ionizing radiation, CT scans must be used opportunistically from routine medical care (15), although CT does allow for high resolution imaging of fat and muscle depots. MRI also allows for high resolution imaging, but without the need for ionizing radiation though is more expensive and contraindicated in some patients with metallic implants or claustrophobia (13, 14). In contrast, US is less expensive, portable, and capable of bedside imaging. Multiple US-based metrics can also be acquired including brightness-mode and shear wave elastography, which images tissue using soundwaves (16, 17).

These imaging methods all use measurements with the potential to be biomarkers that can assess muscle quality, which is recommended over biomarkers assessing muscle mass because muscle strength begins to decrease before muscle mass (3, 7, 18). One cause of decreased muscle quality is infiltration of fat into muscle tissue, or myosteatosis (19). Myosteatosis decreases muscle function, compromises mitochondrial function, and increases the inflammatory response in muscle (19). Therefore, there is a need to quantify myosteatosis in a clinical setting, including in sarcopenia and cachexia diagnoses.

MRI-based chemical-shifted encoded proton density fat fraction (PDFF) is a robust biomarker with high accuracy and repeatability that can quantify percent fat in various tissues including liver, pancreas, and muscle (20–23). CT has also shown promise as a biomarker of myosteatosis by estimating the tissue X-ray attenuation in Hounsfield Units (HU) (24, 25). Lower HU, indicating lower density and higher lipid infiltration, is associated with mortality in numerous patient populations (26–29). US can assess muscle health via brightness mode or shear wave elastography at the bedside. The analysis of pixel brightness, or echo intensity (EI), is associated with fat infiltration in muscle, fibrous tissue, and lower muscle function (30, 31). Shear wave elastography assesses tissue shear wave propagation speed through the relationship between strain and stretch; however, the relationship between muscle shear wave speed and muscle quality is not clear with higher and lower shear wave speed reported in older populations depending on the muscle (14, 32–34). Although US is associated with muscle health, the extent to which US can be used as a measurement of myosteatosis remains unclear, especially to detect differences from aging and disease.

The purpose of this work was to develop biomarkers that can assess the state of muscle health and be used as therapeutic targets repeatedly through time in sarcopenia and cachexia diagnosis and management. We propose using PDFF as a reference of myosteatosis to determine how EI and shear wave speed can be used to measure myosteatosis in aging and disease states. We hypothesize that PDFF is reflective of aging and disease, and EI and shear wave speed are associated with both PDFF and tissue density from CT.

## Materials and Methods

### Subjects

This pilot study and the review of CT scans from health records were approved by the University of Wisconsin-Madison Health Sciences Institutional Review Board. Young and older healthy adults were recruited by advertising on a university email listserv or word of mouth, and were eligible if they had no known significant medical concerns including diabetes, cardiovascular disease, organ transplant, or neuromuscular disease. Oncologists recruited their patients during patient visits to the Carbone Cancer Center at the University of Wisconsin Health Medical Center who were undergoing systemic treatment for non-small cell lung cancer. These patients were eligible if they were deemed healthy enough by their oncologist and did not have uncontrolled diabetes. Groups were selected to ideally capture differences in myosteatosis due to aging (between young and older healthy adults) and disease (between older healthy adults and older adults undergoing treatment).

Exclusion criteria for all participants included any contraindication to MRI identified by routine MRI safety screening. Each potential participant was informed about the study and screened for inclusion and exclusion criteria via telephone. Eligible participants underwent an ~2.5-h study visit that included an informed consent process, height and weight measurements, MRI measurements, brightness-mode ultrasound measurements, shear wave elastography measurements, and a muscle biopsy of the rectus femoris (RF).

### MRI Measurements

Each participant was screened for MRI safety before undergoing any MRI procedures. Participants were scanned while supine using a 3.0T MRI scanner (SIGNA Premier, GE Healthcare, Waukesha, WI) with a 30-channel array AIR anterior receive coil centered over the thigh. Participants underwent single-shot fast spin-echo sequence to orient and localize the mid-thigh and pelvis. A commercial confounder-corrected chemical shift encoded MRI (CSE MRI) method (IDEAL IQ, GE Healthcare) was used to generate PDFF maps of the thigh in both legs. The acquisition was performed using the following parameters: TR = 7.2 ms, 6 echoes in two echo trains of 3 echoes, 3° flip angle, 220 × 220 × 36 matrix, 46 × 46 cm^2^ field of view, 6 mm slice thickness (35).

**Figure 1:**
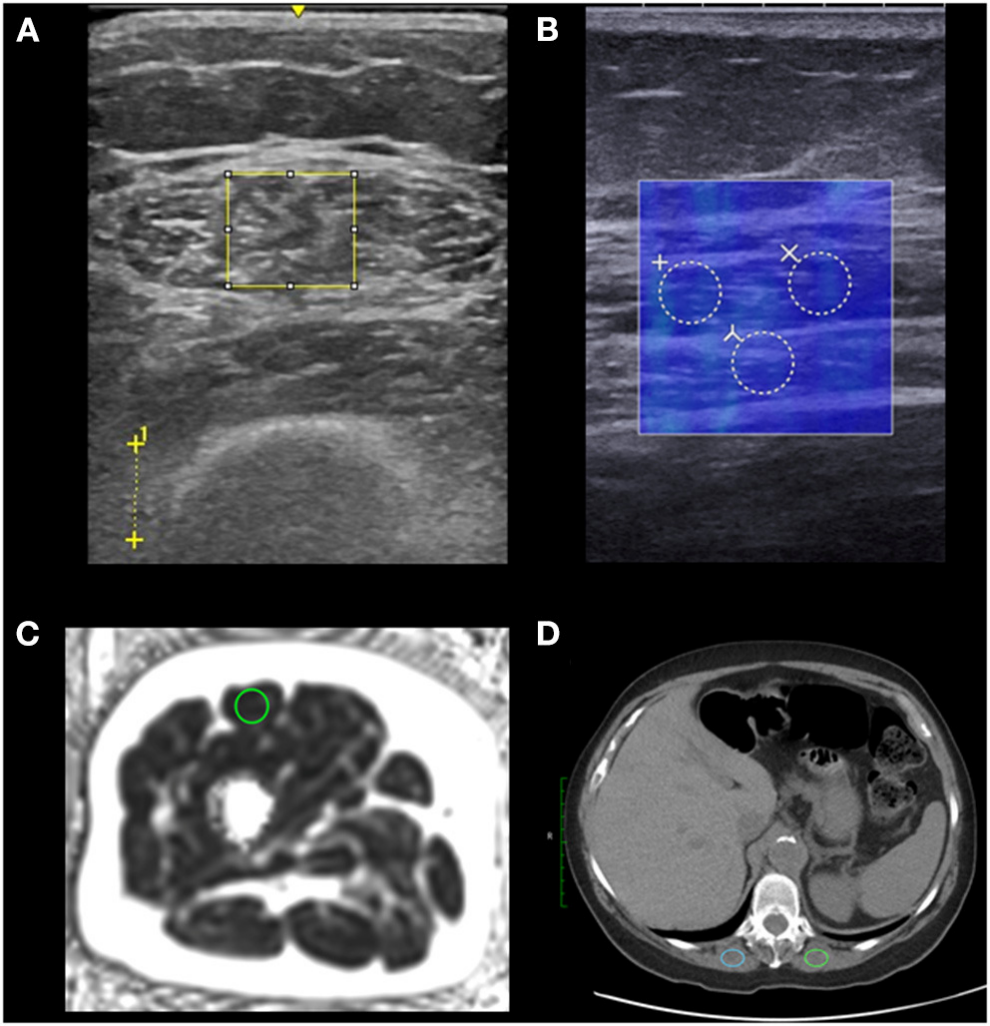
Regions of interest (ROIs) used to analyze myosteatosis. We placed ROIs in the rectus femoris of the right leg using ultrasound and MRI (*n* = 30), and ROIs in the erector spinae muscles using CT for older adults undergoing treatment (*n* = 10). We placed the largest possible rectangular ROIs to assess echo intensity on brightness-mode ultrasound and used the distance between crosses to indicate 1 cm to set image scale for measuring tissue thicknesses in post processing **(A)**. We placed three 3 mm diameter circular ROIs which were averaged to assess shear wave speed measured by shear wave elastography. The shear wave speed measurements (in blue) are coregistered with a brightness-mode image to identify tissue locations **(B)**. We placed a 1 cm^2^ circular ROI to assess proton density fat fraction using MRI **(C)**. We placed 2 ovular 2 cm^2^ ROIs which were averaged to assess the muscle density using CT of older adults undergoing treatment **(D)**.

One trained researcher analyzed the PDFF images that matched quadriceps anatomy of the ultrasound brightness mode images in OsiriX (Pixmeo SARL, Geneva, Switzerland). The researcher measured RF cross-sectional area, whole-leg cross-sectional area, and RF PDFF for each participant. The researcher used circular, 1 cm^2^ regions of interest (ROI) in the RF centrally to obtain RF PDFF values (see Figure 1). We used RF PDFF as the MRI-based measure of myosteatosis.

### Echo Intensity Measurements

Two cross-sectional brightness scale static images per participant were obtained by separate researchers using a LogicE ultrasound machine (GE Healthcare, Waukesha, WI) with a 9 kHz linear transducer probe with gain set to 50 for each image. Each participant laid supine with legs relaxed on a hospital bed. We marked the participant's skin with marker at the point equidistant to the superior iliac crest and the proximal edge of the patella. Excess gel and minimal pressure were used to minimize tissue compression.

One trained researcher with 2 years of musculoskeletal US experience measured RF thickness (cm), subcutaneous adipose tissue thickness (cm), and distance from the skin to the femur (cm) using the LogicE ultrasound machine for both brightness-mode images for each participant. The researcher also measured RF EI in arbitrary units using ImageJ Java version 8 (National Institutes of Health, Bethesda, MD) by adding the largest possible rectangular ROI in the RF while avoiding the central tendon (see Figure 1). The researcher then used the histogram function to calculate the mean pixel intensity within the ROI for each image. The RF EI values of both images per participant were averaged to calculate RF EI for each participant. RF EI served as our myosteatosis measure for brightness-mode ultrasound.

### Shear Wave Elastography Measurements

A trained sonographer with 6 years of musculoskeletal US experience captured static images of the RF using a shear wave elastography capable SuperSonic Imagine Aixplorer (SuperSonic, Hologic, Aix-en-Provence, France) with a Linear SL10-2L transducer (center frequency = 12 MHz) in the musculoskeletal preset. The sonographer captured longitudinal images in parallel with the RF fascicles equidistance between the superior iliac crest and the proximal edge of the patella at the same place as the brightness-mode US static images. The sonographer added 3 circular ROI each with 3 mm diameters in post-processing yielding 3 shear wave speed values in meters per second which were averaged to determine RF shear wave speed (see Figure 1). The shear wave speed of the RF served as our myosteatosis measure for shear wave elastography.

### CT Measurements

Paraspinal CT measurements for each older adult undergoing treatment were obtained from their medical records from routine non-contrast CT of the chest. We used axial images localized to the twelfth thoracic vertebrae (T12) in CT image analyses in Osirix MD (Pixmeo, Bernex, Switzerland). We placed 2 cm^2^ ovoid ROIs in the erector spinae muscles bilaterally and the Hounsfield units (HU) were averaged to obtain erector spinae HU (see Figure 1). If the available erector spinae areas were <2 cm^2^, the largest available region of interest was used. Eight CT scans were non-contrast, and two had early arterial phase contrast. CT scanners used were GE Revolution HD (*n* = 4), GE Revolution EVO (*n* = 3), and GE Optima (*n* = 3) (GE Healthcare, Madison, WI) and all but 1 scan was conducted at the University of Wisconsin Health Hospital in Madison, WI.

### Biopsy and Histology

A musculoskeletal trained radiologist with 19 years of experience and sonographer used the SuperSonic Imagine Aixplorer to guide a 17-gauge co-axial introducer needle (Argon Medical Devices, Athens, Texas) and an 18-gauge needle (BioPince, Argon Medical Devices, Athens, Texas) to collect a single soft tissue biopsy sample of the RF. Biopsy samples were immediately fixed in formalin and set for at least 24 h before dissection and histological analyses.

A pathologist carried out deparaffinization and automated immunohistochemistry on a Ventana Discovery Ultra BioMarker Platform (Roche, Indianapolis, United States), in addition to heat-induced epitope retrieval in the form of “cell conditioning” with CC1 buffer (Ventana #950-500), a Tris based buffer pH 8.4, for ~62 min at 95°C. Slides were incubated with primary antibody mouse-anti-human CD68 (KP-1) for 12 min at 37°C and were rinsed with Reaction Buffer (Ventana #950-300). Discovery Omni-Map anti-Mouse HRP (Ventana #760-4310) was applied for 8 min at 37°C then rinsed with Reaction Buffer (Ventana #950-300). Discovery ChromoMap DAB detection kit (Ventana #760-159) was applied to the slides. For hematoxylin and eosin staining, the slides containing biopsy were counterstained with Harris hematoxylin (VWR, #10143-606) diluted 1:5 with dH2O for 45 s. Slides were rinsed with dH2O, dehydrated, dipped in xylene, and cover slipped using Mounting Medium (Thermo, Cat# 4112). A pathologist graded each sample for variation in muscle fiber size, necrotic fibers via CD68 immunoreactivity, split fibers, and inflammatory infiltrates.

The hematoxylin and eosin-stained slide as well as an immunohistochemically stained slide for CD68 was reviewed by a neuropathologist on every muscle biopsy obtained from individual patients. The neuropathologist was blinded to each participant's group. The amount of internalized nuclei were scored as mild (observed in <10% of fibers; consistent with normal muscle), moderate (observed in 10–30% of fibers), or severe (observed in >30% of fibers). Muscle fiber type variability was scored as mild, moderate, or severe depending on overall heterogeneity in muscle fiber size compared to that generally observed in normal muscle (mild variability). Necrotic fibers, split fibers, and inflammatory infiltrates were also assessed and scored on a scale of none, rare, multifocal, and diffuse. Lipid infiltration within muscle was scored on a scale of none, mild, moderate, or severe. CD68 immunohistochemistry was scored on a scale of none, rare, multifocal, or diffuse.

### Statistical Analyses

We performed all data cleaning and analysis using SAS software (Version 9.4, SAS Institute Inc, Cary, NC, USA). We conducted interrater reliability tests for US EI from the two RF brightness mode images from two researchers using the Shroud and Fleiss ICC (1, 2) calculation (36). We calculated means, standard deviations, and frequencies for each continuous and categorical variable, respectively for the entire sample stratified by participant group. We then tested the normality for each continuous variable using Shapiro-Wilk tests with a *p* < 0.05 indicating non-normality. Because several variables were non-normally distributed, we used Spearman correlations to determine associations between continuous variables with α = 0.05 for the entire sample. We also conducted Spearman correlations among older adults undergoing treatment to examine the associations with continuous variables including paraspinal muscle density. We conducted non-parametric Kruskal-Wallis tests to compare median values between all groups with α = 0.05 and Dwass-Steel-Critchlow-Fligner multiple comparisons *post-hoc* procedures with α = 0.05 to compare median values in 1 vs. 1 group comparisons.

## Results

All descriptive statistics with group comparisons from this pilot study are shown in Table 1. We had a total of 30 participants (15 women) in this study comprised of 10 young healthy adults (ages 18–30, five women), 10 older healthy adults (ages 55–71, five women), and 10 older adults undergoing treatment (ages 49–82, five women). Young adults did not differ in BMI from older healthy adults or older adults undergoing treatment (23.67, 24.51, 27.43 kg/m^2^, respectively).

**Table 1:** Descriptive statistics of each group.

	**Young healthy**			**Older healthy**			**Older cancer**		
	***n***	**Mean**	**SD**	***n***	**Mean**	**SD**	***n***	**Mean**	**SD**
Age (years)	10	22.80^A^	3.82	10	61.70^B^	5.46	10	61.40^B^	9.56
BMI (kg/m^2^)	10	23.67	3.66	10	24.51	4.67	10	27.43	6.56
RF Thickness (cm)	10	2.26^A^	0.44	10	1.79^A, B^	0.45	10	1.70^B^	0.37
SAT Thickness (cm)	10	0.93	0.52	10	1.25	0.67	10	1.08	0.68
RF Echo Intensity (A.U.)	10	48.58^A^	20.58	10	81.81^B^	27.20	10	75.35^A, B^	28.68
RF Shear wave speed (m/s)	10	1.86	0.75	10	1.74	0.77	10	1.74	0.89
RF CSA (cm^2^)	10	5.72	2.31	10	4.80	1.45	10	4.45	1.21
Whole-leg CSA (cm^2^)	10	208.18	39.55	10	203.23	41.83	10	195.63	63.62
Right Leg RF PDFF (%)	10	0.33^A^	0.66	10	2.83^B^	3.27	10	2.93^B^	2.44

*Differences in groups are indicated by letter superscript with no letters or shared letters indicating no significant differences and with unshared letters between groups indicating significant median differences (p < 0.05) based on Kruskal-Wallis tests and Dwass-Steel-Critchlow-Fligner multiple comparisons post-hoc procedures. A.U., arbitrary units; BMI, body mass index; CSA, cross-sectional area; PDFF, proton density fat fraction; RF, rectus femoris; SAT, subcutaneous adipose tissue; SD, standard deviation*.

PDFF differed significantly between groups (X^2^ = 9.130; *p* = 0.0104) with young adults having significantly lower PDFF values than both older healthy adults (Wilcoxon *Z* = −2.6458, *p* = 0.0304) and older adults undergoing treatment (Wilcoxon *Z* = 0.378, *p* = 0.9243), but older healthy adults and older adults undergoing treatment did not differ in PDFF (Wilcoxon *Z* = 0.3780, *p* = 0.9243). Interrater reliability of US EI between the two operators' images was very high at 0.956. RF EI also differed significantly between groups (X^2^ = 8.3226, *p* = 0.0156) with young healthy adults having significantly lower EI than older healthy adults (Wilcoxon *Z* = −2.6458, *p* = 0.0222) but not significantly lower EI than older adults undergoing treatment (Wilcoxon *Z* = −2.2678, *p* = 0.0604). Older healthy adults and older adults undergoing treatment did not differ in RF EI (Wilcoxon *Z* = −0.3780, *p* = 0.9243). RF thickness similarly differed significantly between groups (X^2^ = 7.9718, *p* = 0.0186) with young healthy adults having significantly higher RF thickness than older healthy adults (Wilcoxon *Z* = 2.5711, *p* = 0.0222) but not significantly higher RF thickness than older adults undergoing treatment (Wilcoxon *Z* = 2.1947, *p* = 0.0721). Older healthy adults and older adults undergoing treatment did not differ in RF thickness (Wilcoxon *Z* = −0.6047, *p* = 0.8175). Rectus femoris shear wave speed, subcutaneous adipose tissue thickness, rectus femoris cross-sectional area, BMI, or whole-leg cross-sectional area did not differ between groups.

When examining bivariate associations (Table 2), RF PDFF was strongly associated with RF EI (ρ = 0.753, *p* < 0.0001) and SAT thickness (ρ = 0.556, *p* = 0.0015), moderately negatively associated with RF shear wave speed (ρ = −0.489, *p* = 0.0060), weakly negatively association with RF thickness (ρ = −0.378, *p* = 0.0393) (37). PDFF was not associated with RF CSA, whole leg CSA, nor BMI. RF EI was positively associated with SAT thickness (ρ = 0.754, *p* < 0.0001), and negatively RF thickness (ρ = −0.548, *p* = 0.0017) and RF CSA (ρ = −0.398, *p* = 0.0292). BMI was only associated with whole-leg cross sectional area (ρ = 0.386, *p* = 0.0354) and not SAT thickness nor PDFF. When stratifying among older adults undergoing treatment who had CT scans, paraspinal muscle density was strongly associated with BMI (ρ = −0.854, *p* = 0.0016) and PDFF (ρ= −0.696, *p* = 0.0251; Table 3). The mean time between the study visit and the most recent non-contrast chest CT scan was 11.4 ± 9.2 days.

**Table 2:** Bivariate associations between myosteatosis and adiposity measures among the entire sample (*n* = 30).

**Measure**	**RF thickness**	**RF EI**	**RF shear wave speed**	**RF CSA**	**BMI**	**RF SAT thickness**	**Whole-leg CSA**	**RF PDFF**
RF thickness	1	−0.548**	0.1967	0.636***	−0.138	−0.374*	0.260	−0.378*
RF EI	−0.548**	1	−0.322	−0.398	0.359	0.754***	0.259	0.753***
RF shear wave speed	0.197	−0.322	1	−0.02	−0.099	−0.123	0.013	−0.489**
RF CSA	0.636***	−0.398*	−0.02	1	−0.042	−0.385*	0.359	−0.321
BMI	−0.138	0.3592	−0.099	−0.042	1	0.122	0.386*	0.269
RF SAT thickness	−0.374*	0.754***	−0.123	−0.385	0.1221	1	0.408*	0.553**
Whole-leg CSA	0.2699	0.2591	0.013	0.359	0.386*	0.408*	1	0.211
RF PDFF	−0.378*	0.753***	−0.489**	−0.321	0.269	0.556**	0.211	1

*BMI, body mass index; CSA, cross-sectional area; EI, echo intensity; PDFF, proton density fat fraction; RF, rectus femoris; SAT, subcutaneous adipose tissue*;

^*^p < 0.05,

^**^p < 0.01,

^***^p < 0.001.

**Table 3:** Bivariate associations between myosteatosis, including CT scans, and adiposity measures among older adults undergoing treatment for lung cancer (*n* = 10).

**Measure**	**RF thickness**	**RF EI**	**RF shear wave speed**	**RF CSA**	**BMI**	**SAT thickness**	**Whole-leg CSA**	**RF PDFF**	**Paraspinal muscle density**
RF thickness	1	−0.151	−0.249	0.6363	0.4909	0.0787	0.3212	0.0909	−0.187
RF EI	−0.151	1	−0.182	−0.478	0.3575	0.8909***	0.6242	0.709*	−0.406
RF shear wave speed	−0.249	−0.182	1	−0.079	−0.656*	−0.176	−0.455	−0.522	0.5106
RF CSA	0.6363*	−0.478	−0.079	1	0.296	−0.406	0.296	−0.296	0.0909
BMI	0.4909	0.3575	−0.656*	0.296	1	0.4545	0.6121	0.6606*	−0.854**
RF SAT thickness	0.0787	0.8909***	−0.176	−0.406	0.4545	1	0.5515	0.6484*	−0.43
Whole-leg CSA	0.3212	0.6242	−0.455	0.296	0.6121	0.5515	1	0.5272	−0.309
RF PDFF	0.0909	0.709*	−0.522	−0.296	0.6606*	0.6484*	0.5272	1	−0.696*
Paraspinal muscle density	−0.187	−0.406	0.5106	0.0909	−0.854**	−0.43	−0.309	−0.696*	1

*BMI, body mass index; CSA, cross-sectional area; EI, echo intensity; PDFF, proton density fat fraction; RF, rectus femoris; SAT, subcutaneous adipose tissue*;

^*^p < 0.05,

^**^p < 0.01,

^***^p < 0.001.

The histological analyses and grading of rectus femoris biopsy samples between older healthy adults and adults undergoing treatment show no differences in any analyses (Table 4).

**Table 4:** The results of histological analyses of rectus femoris biopsies.

**Method**	**Pathology grading**	**Older healthy adults**	**Adults undergoing treatment**
Internalized nuclei	Mild	2*	2
	None	7*	8
Muscle fiber variability	Mild	9*	10
Necrotic fibers	None	9*	10
Lipid infiltration	None	9*	10
Split fibers	None	9*	10
Inflammatory infiltrate	Not Present	10	10
CD68	Negative	10	10

*Group frequencies and grading are essentially identical*.

^*^n = 9 instead of 10 due to technical error.

## Discussion

In these results from a pilot study, we demonstrated that RF EI was strongly associated with RF PDFF, which suggests EI has the potential be used as a biomarker of myosteatosis. We also found that RF PDFF was significantly associated with paraspinal muscle density. RF CSA and RF thickness, both previously used proxies of muscle mass, were either not associated or weakly associated with RF PDFF, respectively. When comparing groups, we also found that PDFF was significantly lower among young healthy adults compared to older healthy adults and older adults undergoing treatment.

Our results support previous findings of increased myosteatosis with aging and recapitulate the need for myosteatosis and muscle quality biomarkers instead of muscle mass-based biomarkers (3). Our findings also support the need to use muscle-based biomarkers to assess muscle health instead of whole-leg CSA or especially BMI, as BMI was not associated with PDFF, EI, shear wave speed, RF CSA, or RF thickness. Our results demonstrate an opportunity to use muscle quality imaging biomarkers to simultaneously assess muscle health for diagnostic purposes and evaluate therapeutic targets, both of which may lead to improved patient outcomes.

Although we did not find significant PDFF differences between the older adult groups, PDFF still may be a useful biomarker to detect sarcopenia and cachexia progression among patients with cancer especially as populations become more obese and remain on longer treatment regimens (38). PDFF may prove beneficial as a repeatable biomarker because of its almost identical quantification of triglycerides by magnetic resonance spectroscopy (21). Although magnetic resonance spectroscopy is highly accurate when quantifying intra- and extramyocellular lipid species, it requires specialized software to acquire and specialized expertise to analyze (39). Percent fat has also been quantified from MRI using T1-weighted images; however, Akima et al. determined T1-weighted estimation of percent fat primarily captures extramyocellular lipids and not intramyocellular lipids as measured by magnetic resonance spectroscopy (40). PDFF is a fundamental property of tissue that reflects the true concentration of triglycerides, independent of intra- or extramyocellular location, and is highly repeatable and reproducible (20, 41). PDFF also has the advantage over spectroscopy in a clinical setting, because clinicians can assess large, adjustable ROIs across volumetric data (20, 21, 39).

The non-significant differences between older adults undergoing treatment and older healthy adults when comparing EI and PDFF may be explained by the short time from starting systemic treatment. Non-significant EI differences but significant PDFF differences among older adults undergoing treatment and young healthy adults may be explained by the types of lipids captured by US and PDFF. Akima et al. also showed that EI only captured extramyocellular lipids (40) unlike PDFF which captures both intra- and extramyocellular lipids possibly leading to a mismatch of which lipids are captured by each imaging method (21). Given the convenience and availability of EI analysis, technicians and physicians could assess muscle at the bedside if patients are non-ambulatory or non-responsive without radiation or contraindication to magnetic metals (13). PDFF, and especially EI, may still serve as repeatable assessments of muscle health that can be targeted for specific muscles. Our results show EI and PDFF reflect differences in aging better than BMI and may serve as better indicators of muscle wasting.

Shear wave speed was moderately negatively associated with PDFF. However, shear wave speed was not significantly different between groups but trended in the hypothesized direction of lower shear wave speed among older healthy adults and older adults undergoing treatment when compared to young healthy adults. These results are consistent with another study on shear wave speed and the stages of shoulder muscle fat infiltration (42). However, other studies have found conflicting results regarding shear wave elastography and age, perhaps due to various muscle group differences (14). Eby et al. found that muscle stiffness, another quality indicator determined by shear wave elastography, tended to increase with age in the biceps brachii while Akagi et al. found higher elasticity in younger participants in the rectus femoris and the gastrocnemius (33, 43). When examining supraspinatus muscle, Rosskopf et al. found higher lipid concentration was associated with lower shear wave velocity which is consistent with our findings (42). Therefore, more research is needed to determine if shear wave elastography can adequately evaluate muscle health and is sensitive to myosteatosis.

CT-based paraspinal muscle density was positively associated with BMI and negatively associated with RF PDFF. This association with BMI corresponds with previous research (44). PDFF is expected to be inversely associated with muscle density as increased lipid infiltration within muscle lowers tissue HU (45). Similar to our findings, Faron et al., found a high correlation between CT-based paraspinal muscle density and MRI-based paraspinal PDFF among patients with cancer with the recommendation that both measures could be used interchangeably (46). To avoid the use of ionizing radiation in study participants, only the opportunistic use of chest CT scans were analyzed among older adults undergoing treatment. Therefore, analogous muscle groups were not assessed between US and CT, and CT and MRI, which would likely increase associations between measures.

The older healthy adults and older adults undergoing treatment showed almost identical histology analyses and grading for internalized nuclei, muscle fiber variability, necrotic fibers, lipid infiltration, split fibers, inflammatory infiltrate, and CD68 staining. Our 18-gauge needle biopsy may limit the amount of tissue collected; however, these results support that biopsies may not reflect pathologies because sampling error (47). Biopsy is seen as the “gold standard” for tissue composition assessment, yet invasive biopsies sample only a tiny fraction of a heterogenous organ (47). As an example, estimates of liver biopsies sample only 1/50,000th of the entire liver (47). Therefore, non-invasive imaging methods are needed that can assess myosteatosis at multiple points of muscle repeatedly through time can be used as both diagnostic biomarkers and therapeutic targets.

Although our results show promise, our study has several limitations. Because the study is a pilot, the small sample size may be masking true differences between groups. Our healthy adult sample was a convenience sample of adults on a university campus, which may not reflect a general population. Our participants with cancer were recruited toward the beginning of their treatment, which may not reflect changes in muscle health or overall health during the average length of treatment. In addition, the older adults undergoing treatment were not confirmed to have cachexia or sarcopenia. We also did not have initial treatment start dates for all participants. Of the 10 older adults undergoing treatment, nine self-reported their time since beginning treatment, all of whom started treatment within a year or less of their study visit. We also did not assess muscle function nor diet, which are both associated with mortality (3, 48).

Our study does have several strengths including comparing MRI-based PDFF, US-based EI, US-based shear wave speed, CT-based density, and histology in muscle among different age groups and among older adults undergoing treatment. Barring sample size, we were able to show age differences among PDFF and EI and show both measures are markers of myosteatosis with the potential for clinical relevance, unlike BMI, SAT thickness, or whole-leg CSA. We also show that not all muscle quality measures are equally associated with one another, and that some may lend themselves to be better measures of muscle quality than others.

More research will be needed to compare PDFF and our US measures to CT, dual-energy x-ray absorptiometry, and bioimpedance analysis to muscle function in larger samples, especially patients undergoing treatment for lung cancer. The addition of muscle mitochondrial function would also greatly enhance the understanding of what the imaging techniques may capture when assessing muscle (49).

## Conclusion

In our results from this pilot study, we demonstrated that EI and shear wave speed are associated with PDFF, supporting their use as potential biomarkers of myosteatosis given more research. RF thickness and RF CSA were not associated with PDFF suggesting traditional markers of muscle mass do not reflect changes in myosteatosis and thus muscle quality. PDFF and EI have the potential to become measures of muscle health and intervention targets for diseases like sarcopenia and cachexia. More research is needed to determine the timepoints of when myosteatotic, and therefore muscle quality, changes occur and become pathologic in sarcopenia and cachexia, and how PDFF and EI may relate to other imaging techniques and cellular function.

## Data Availability Statement

The raw data supporting the conclusions of this article will be made available by the authors, without undue reservation.

## Ethics Statement

The studies involving human participants were reviewed and approved by University of Wisconsin-Madison Health Sciences Institutional Review Board. The patients/participants provided their written informed consent to participate in this study.

## Author Contributions

JL: conceptualization, recruitment, data curation, methodology, data analysis, writing original draft, writing review and editing, and project management. BR: conceptualization, recruitment, data curation, methodology, data analysis, statistical analysis, data visualization, writing original draft, writing review and editing, and project management. KO: conceptualization, recruitment, data curation, methodology, and writing review and editing. TJC: conceptualization, data curation, methodology, data analysis, and writing review and editing. DT: data curation, data analysis, and writing review and editing. SG: data analysis and writing review and editing. TCC: recruitment and writing review and editing. AT: recruitment and writing review and editing. TL: conceptualization, recruitment, methodology, and writing review and editing. KL and SR: conceptualization, data curation, methodology, writing review and editing, and funding acquisition. AK: conceptualization, recruitment, data curation, methodology, writing original draft, writing review and editing, funding acquisition, and project management supervision. All authors contributed to the article and approved the submitted version.

## Funding

BR was supported in part by the National Institute of Food and Agriculture, United States Department of Agriculture Hatch project (1023263). KO was supported in part by the National Institute of Diabetes and Digestive and Kidney Diseases (T32DK007665). TJC and SR were supported by the National Institute of Diabetes and Digestive and Kidney Diseases (R01 DK088925 and K24 DK102595) and the National Institute of Health (R01 DK100651). TCC was supported by the Ellen and Peter O. Johnson Chair in Palliative Care (UW-Foundation 132580106). KL was supported by the RSNA Scholar Grant RSCH1317, the University of Wisconsin Madison Radiology Department Research and Development Fund (#1204-001), and the Clinical and Translational Science Award (CTSA) program, previously through the National Center for Research Resources (NCRR) grant 1UL1RR025011, and now by the National Center for Advancing Translational Sciences (NCATS), grant 9U54TR000021. AK was supported by the Clinical and Translational Science Award (CTSA) program through the National Center for Advancing Translational Sciences (NCATS), grants UL1TR002373 and KL2TR002374.

## Conflict of Interest

The authors declare that the research was conducted in the absence of any commercial or financial relationships that could be construed as a potential conflict of interest.

## Publisher's Note

All claims expressed in this article are solely those of the authors and do not necessarily represent those of their affiliated organizations, or those of the publisher, the editors and the reviewers. Any product that may be evaluated in this article, or claim that may be made by its manufacturer, is not guaranteed or endorsed by the publisher.
