# Corrigendum: Myosteatosis as a Shared Biomarker for Sarcopenia and Cachexia Using MRI and Ultrasound

**DOI:** 10.3389/fresc.2022.982949

**Published:** 2022-07-15

**Authors:** Jevin Lortie, Benjamin Rush, Katie Osterbauer, T. J. Colgan, Daiki Tamada, Sujay Garlapati, Toby C. Campbell, Anne Traynor, Ticiana Leal, Viharkumar Patel, Jeffrey J. Helgager, Kenneth Lee, Scott B. Reeder, Adam J. Kuchnia

**Affiliations:** ^1^Department of Nutritional Sciences, University of Wisconsin-Madison, Madison, WI, United States; ^2^Department of Radiology, University of Wisconsin-Madison School of Medicine and Public Health, Madison, WI, United States; ^3^Department of Medicine, University of Wisconsin-Madison School of Medicine and Public Health, Madison, WI, United States; ^4^Department of Hematology and Medical Oncology, Emory University School of Medicine, Atlanta, GA, United States; ^5^Department of Pathology, Harvard Medical School, Boston, MA, United States; ^6^Department of Pathology, University of Wisconsin-Madison School of Medicine and Public Health, Madison, WI, United States

**Keywords:** proton density fat fraction, echo intensity, ultrasound, MRI, cancer, elasticity, muscle quality, muscle health

In the published article, there was an error in the author list, and author “Viharkumar Patel” and “Jeffrey J. Helgager” were erroneously excluded. The corrected author list appears below.

Jevin Lortie^1*†^, Benjamin Rush^1†^, Katie Osterbauer^1^, T. J. Colgan^2^, Daiki Tamada^2^, Sujay Garlapati^1^, Toby C. Campbell^3^, Anne Traynor^3^, Ticiana Leal^3,4^, Viharkumar Patel^5^, Jeffrey J. Helgager^6^, Kenneth Lee^2^, Scott B. Reeder^2^, Adam J. Kuchnia^1^

^1^ Department of Nutritional Sciences, University of Wisconsin-Madison, Madison, WI, United States

^2^ Department of Radiology, University of Wisconsin-Madison School of Medicine and Public Health, Madison, WI, United States

^3^ Department of Medicine, University of Wisconsin-Madison School of Medicine and Public Health, Madison, WI, United States

^4^ Department of Hematology and Medical Oncology, Emory University School of Medicine, Atlanta, GA, United States

^5^ Department of Pathology, Harvard Medical School, Boston, MA, United States

^6^ Department of Pathology, University of Wisconsin-Madison School of Medicine and Public Health, Madison, WI, United States

The authors apologize for this error and state that this does not change the scientific conclusions of the article in any way. The original article has been updated.

## Publisher's note

All claims expressed in this article are solely those of the authors and do not necessarily represent those of their affiliated organizations, or those of the publisher, the editors and the reviewers. Any product that may be evaluated in this article, or claim that may be made by its manufacturer, is not guaranteed or endorsed by the publisher.

